# The Single-Particle, Clusters and Biomolecules and Serial Femtosecond Crystallography instrument of the European X-ray Free-Electron Laser: Interaction Region Downstream at atmospheric pressure (IRD)

**DOI:** 10.1107/S1600577525008999

**Published:** 2026-01-01

**Authors:** Adam Round, Piere Aller, Richard J. Bean, Johan Bielecki, Agata Butryn, Nicholas Devenish, Raphael de Wijn, Thomas Dietze, Katerina Doerner, Fabio Dall’Antonia, Pontus Fischer, Gabriele Giovanetti, Sebastian Guenther, Huijong Han, Vincent Hennicke, Chan Kim, Yoonhee Kim, Marco Kloos, Jayanath C. P. Koliyadu, Gabriel Leen, Romain Letrun, Luis Lopez Morillo, Allen M. Orville, Tim Pakendorf, Marco Ramilli, Nadja Reimers, Patrick Reinke, Juan Sanchez-Weatherby, Tokushi Sato, Robin Schubert, Joachim Schulz, Cedric Signe Takem, Marcin Sikorski, Prasad Thute, Fabian Trost, Oleksii Turkot, Patrik Vagovič, Mohammad Vakili, Raul Villanueva Guerrero, Henry N. Chapman, Alke Meents, Serguei Molodtsov, Sakura Pascarelli, Thomas Tschentscher, Adrian P. Mancuso

**Affiliations:** a European XFEL Holzkoppel 4 22869Schenefeld Germany; b Diamond Light Source Diamond House, Harwell Science and Innovation Campus DidcotOX11 0DE United Kingdom; c Research Complex at Harwell Rutherford Appleton Laboratory DidcotOX11 0FA United Kingdom; d Center for Free Electron Laser Science CFEL Deutsches Elektronen-Synchrotron DESY Notkestrasse 85 22607Hamburg Germany; e PolyPico Technologies Ltd, Unit 10, Airways Technology Park, Rathmacullig West, Ballygarvan, CorkT12 DY95, Ireland; f Department of Electronic and Computer Engineering University of Limerick Limerick Ireland; g Institute of Experimental Physics TU Bergakademie Freiberg Leipziger Strasse 23 09599Freiberg Germany; h Center for Efficient High Temperature Processes and Materials Conversion (ZeHS) TU Bergakademie Freiberg Winklerstrasse 5 09599Freiberg Germany; i Department of Chemistry and Physics, La Trobe Institute for Molecular Science La Trobe University Melbourne Victoria3086 Australia; RIKEN SPring-8 Center, Japan

**Keywords:** X-ray free-electron lasers, XFELs, crystallography, femtosecond experiments

## Abstract

The Single-Particle, Clusters and Biomolecules and Serial Femtosecond Crystallography (SPB/SFX) scientific instrument at the European X-Ray Free-Electron Laser (EuXFEL) became operational with user experiments in September 2017. The unique properties and capabilities of the EuXFEL, enabling megahertz data collection rates, provide more rapid data collection with improved statistics compared with other XFEL facilities. This improves the feasibility of obtaining multiple data points in time-resolved experiments and hence enables the observation of reactions in greater detail (molecular movies). In collaboration with the SFX User Consortium (SFX UC), the SPB/SFX instrument was designed to further increase user access and research outcomes. Focusing the pulses downstream of the first interaction region [described previously (Mancuso *et al.*, 2019)], a second experiment plane is enabled, which allows for greater optimization and more efficient usage of available beam time. Additionally, the SFX UC provided further instrumentation to provide improved capabilities on SPB/SFX. The aim for additional and extended functionality for the second interaction region was to enable sample-efficient data collection at atmospheric pressure in an environment where the sample temperature and humidity can be controlled. This paper describes the extended capabilities of the downstream interaction region of the SPB/SFX instrument and its major components, in particular its X-ray focusing optics, vacuum to atmospheric pressure out-coupling, available sample delivery methods and 2D detector, and the supporting optical laser systems for pump–probe experiments.

## Abstract 2

An introduction is provided to the extended (atmospheric pressure) capabilities of the Single-Particle, Clusters and Biomolecules and Serial Femtosecond Crystallography (SPB/SFX) scientific instrument at the European X-ray Free-Electron Laser facility.

## 1. Introduction

The European X-ray Free-Electron Laser (EuXFEL), the first high-repetition-rate hard X-ray FEL in the world (Abela *et al.*, 2006 ▸), hosts a suite of seven scientific instruments. Each instrument is designed to pursue a wide range of science applications exploiting ultrafast dynamics using ultrabright pulses, as well as uniquely utilizing the megahertz (MHz) peak repetition rate of this unprecedented facility (Tschentscher *et al.*, 2017 ▸). The Single-Particle, Clusters and Biomolecules and Serial Femtosecond Crystallography (SPB/SFX) instrument (Mancuso *et al.*, 2019 ▸) is primarily concerned with 3D structure determination of both crystalline and non-crystalline micrometre-scale and smaller objects. A particular emphasis is placed on biological objects, including viruses, biomolecules and macromolecular crystals.

The popularity and availability of serial crystallography (SX) has increased, as shown by SX now being offered as a standard option at nearly all synchrotron sources around the world. However, the limited availability of beam time at XFELs for serial femtosecond crystallography (SFX) is a major constraint. In order to facilitate SFX experiments at the European XFEL, an additional interaction region has been implemented, encompassing all required equipment and instrumentation for data collection.

The Interaction Region Downstream (IRD) can accommodate a variety of user-designed instrumentation. However, to maximize usage and minimize installation overheads, standard options of SFX experiments are provided. The additional IRD instrumentation to enable efficient SFX operation has been contributed by the SFX User Consortium (SFX, 2013 ▸).

The IRD gives users increased flexibility in their choice of sample delivery (liquid injection, fixed targets) and facilitates studies for temperature- and pressure-sensitive samples. Access to longer timescales enables access to later transient intermediates of multi-stage reactions which require extended incubation times (Fuller *et al.*, 2017 ▸; Kern *et al.*, 2018 ▸; Rabe *et al.*, 2021 ▸; Butryn *et al.*, 2021 ▸). Additional scope for developments of novel concepts for sample delivery is also possible (without the complication of vacuum compatibility).

The goal of this paper is to describe the additional capabilities of the SPB/SFX instrument offered by the ability to undertake experiments at atmospheric pressure, as well as future capabilities which will be facilitated by the downstream interaction region. This paper primarily documents the standard SFX configuration of IRD as commissioned in collaboration with the SFX User Consortium (SFX, 2013 ▸), as used for external user experiments.

## 2. Instrumentation for the downstream interaction region

The SPB/SFX instrument is designed to operate at photon energies from 6 to 24 keV, the details of which have been described previously (Mancuso *et al.*, 2019 ▸). The science requirements and scope of the instrument as published in the SPB Technical Design Report (TDR) (Mancuso *et al.*, 2013 ▸) have not been fundamentally altered, but they have been updated to include the possibility to measure biological samples at atmospheric pressure.

Updated instrument parameters are shown in Table 1 ▸ for reference.

**Figure 1:**
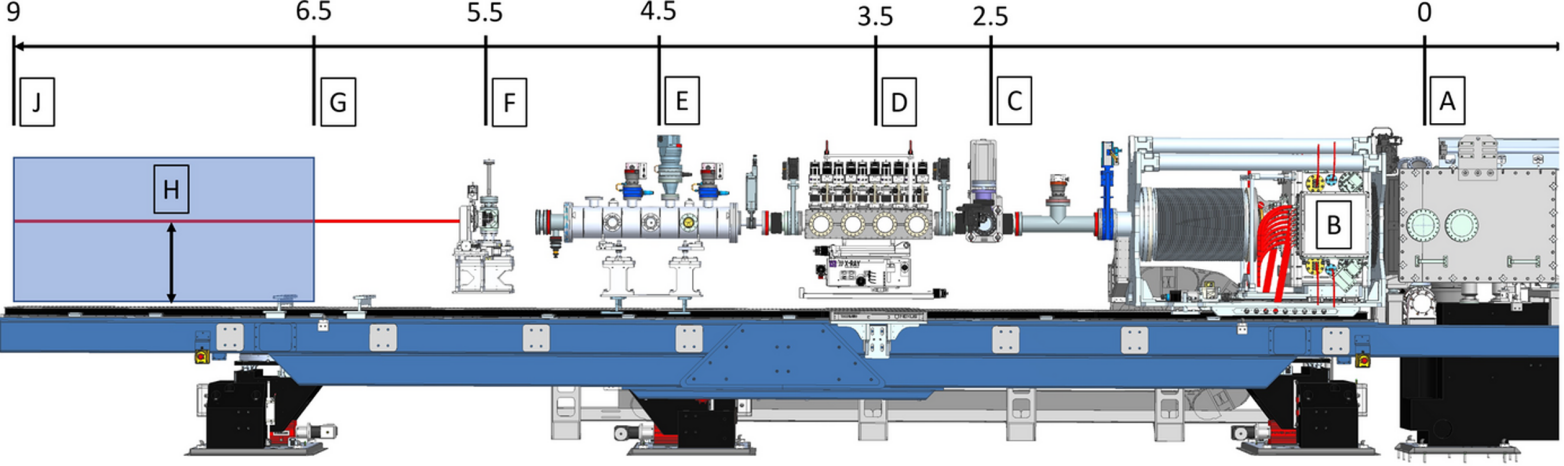
IRD instrumentation installed on the CSS downstream of the IRU. The beam direction is from right to left. Distances are in metres from the IRU focal plane. Components are labelled as follows: A the IRU (focal plane defined as reference point 0 m), B the AGIPD (1M) detector, C the in-vacuum beam visualization screen, D the compound refractive lens transfocator, E the acoustic delay line, F the helium beam visualization screen, G the upstream focus limit, J the downstream focus limit and H an open space for user installations. The region from downstream of the acoustic delay line (E) can be used to install any custom sample environment and/or available detector within the given space constraints (beam height from CSS breadboard = 0.54 m).

The instrumentation necessary for the operation of the IRD (refocusing optics, vacuum to air interface, screens, sample environment and detector) can be installed upon the component support structure (CSS) which facilitates longitudinal positioning along the beam axis using rails (Fig. 1 ▸). Additionally, the optimized SFX sample environment and JUNGFRAU 4M (JF4M) detector are now permanently installed on a dedicated granite support (Fig. 4) designed with the future AGIPD 4M detector in mind.

### 2.1. Refocusing to the downstream interaction region

To increase the throughput of the SPB/SFX instrument, we developed an optimized design implementing X-ray re­focusing [similar to the CXI instrument at LCLS (Boutet *et al.*, 2015 ▸; Hunter *et al.*, 2016 ▸)]. The spot size must be able to match the expected size range of crystals optimally at focal lengths across the IRD to maintain flexibility for installations. The refocusing scheme must also maintain a high fluence to enable observation of diffraction from small crystals. In practice, we aimed to achieve a spot size of a few micrometres, which can be achieved for installations on the downstream end of the CSS 7.5 m from the Interaction Region Upstream (IRU) and the nominal IRD focal plane (9 m from the IRU).

The compound refractive lenses (CRLs) (purchased from RXOPTICS GmbH & Co. KG, Germany) are housed in the dedicated transfocator units described previously (Mancuso *et al.*, 2019 ▸), mounted on the CSS [Fig. 1 ▸(*d*)]. Fine tuning of the focal spot can be achieved either by adjustment of the photon energy of the SASE1 source or by using motorized translation of the transfocator along the optical axis. The CRLs are organized in a binary configuration to facilitate changing the number of lenses in the beam by a single lens. Given the beam size at the position of the transfocator of 800 µm, lenses with radius 300 µm and an acceptance aperture of 1 mm were chosen. A total of 55 lenses with 300 µm radius are installed across seven arms (1, 2, 4, 8, 8, 16, 16), and an additional weaker focusing lens with a radius of 500 µm is installed to facilitate finer adjustment of the focus. The first and last arms of the transfocator unit contain pinholes 2 mm in diameter to aid alignment of the chamber with the X-ray axis.

Either of two focusing schemes can be used in the IRD, focusing the direct beam from the horizontal offset mirrors or refocusing from the spot produced by the micrometre-scale SPB/SFX Kirkpatrick–Baez (KB) mirror system (Bean *et al.*, 2016 ▸), 23 m from the IRU focal plane and 894 m from the SASE-1 source point. The refocusing option requires more CRLs overall (due to the higher divergence of the close secondary source) but provides flexibility for use of the IRD with different instrument configurations.

**Figure 2:**
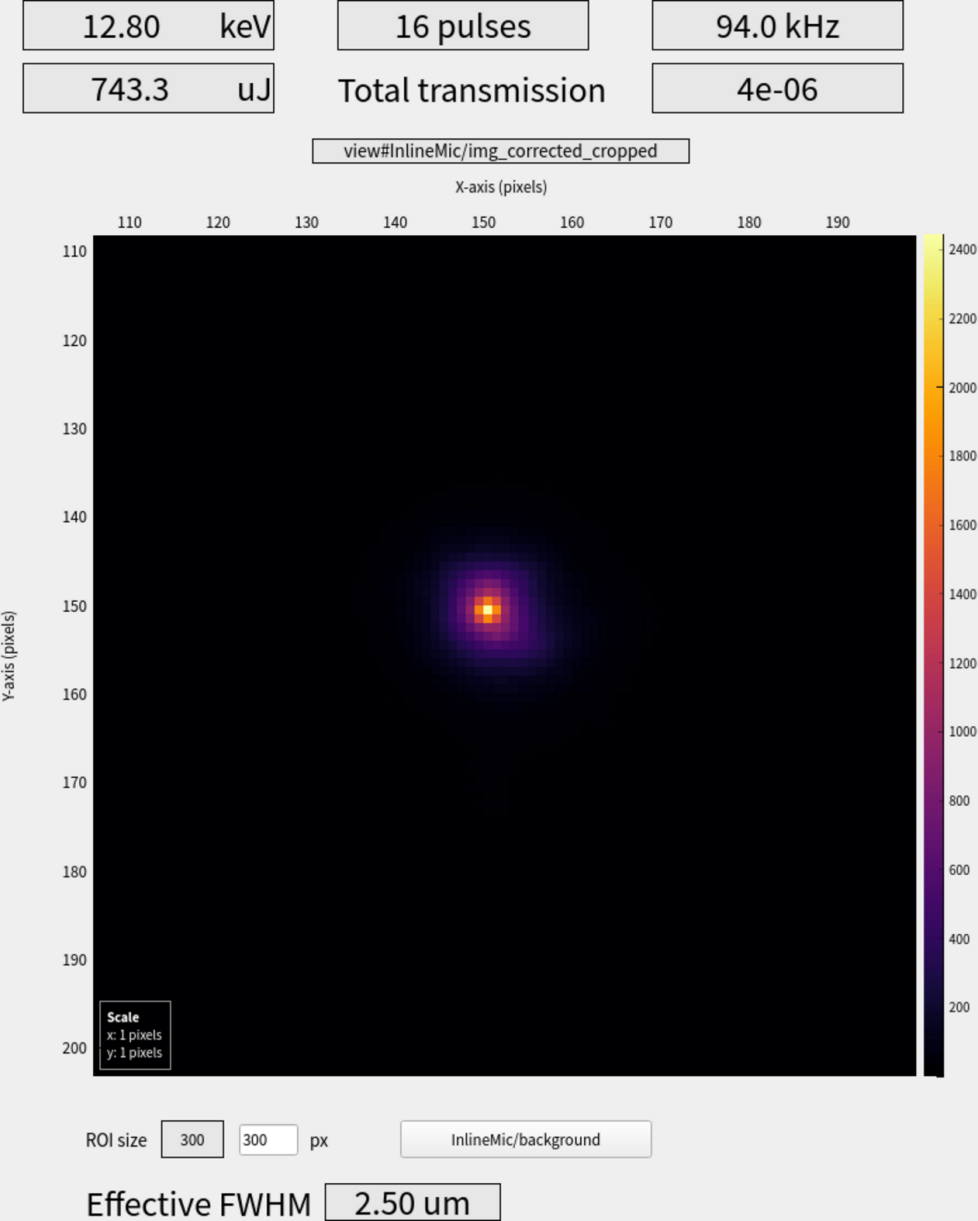
Image from *Karabo* focusing scene used to validate and optimize focus during alignment at the start of each user shift. The focal spot is only a few pixels with 1.042 µm per pixel. The measured effective FWHM (provided in *Karabo* to enable optimization) of the IRD focal spot is routinely ∼2.50 µm (in this case with 12.8 keV X-ray energy) using the direct beam trajectory. A similar effective FWHM is also achievable in the IRD for the refocused micrometre KB trajectory.

The direct beam is focused to the IRD using prefocusing CRLs mounted in the photon beam tunnel (700 m upstream of the SPB/SFX instrument) to match the beam size with the acceptance aperture of the instrument CRLs, thus maximizing fluence. During routine operation, the CRLs focus the X-ray beam, providing a beam spot down to ∼2.5 µm diameter FWHM in the IRD (Fig. 2 ▸).

**Figure 3:**
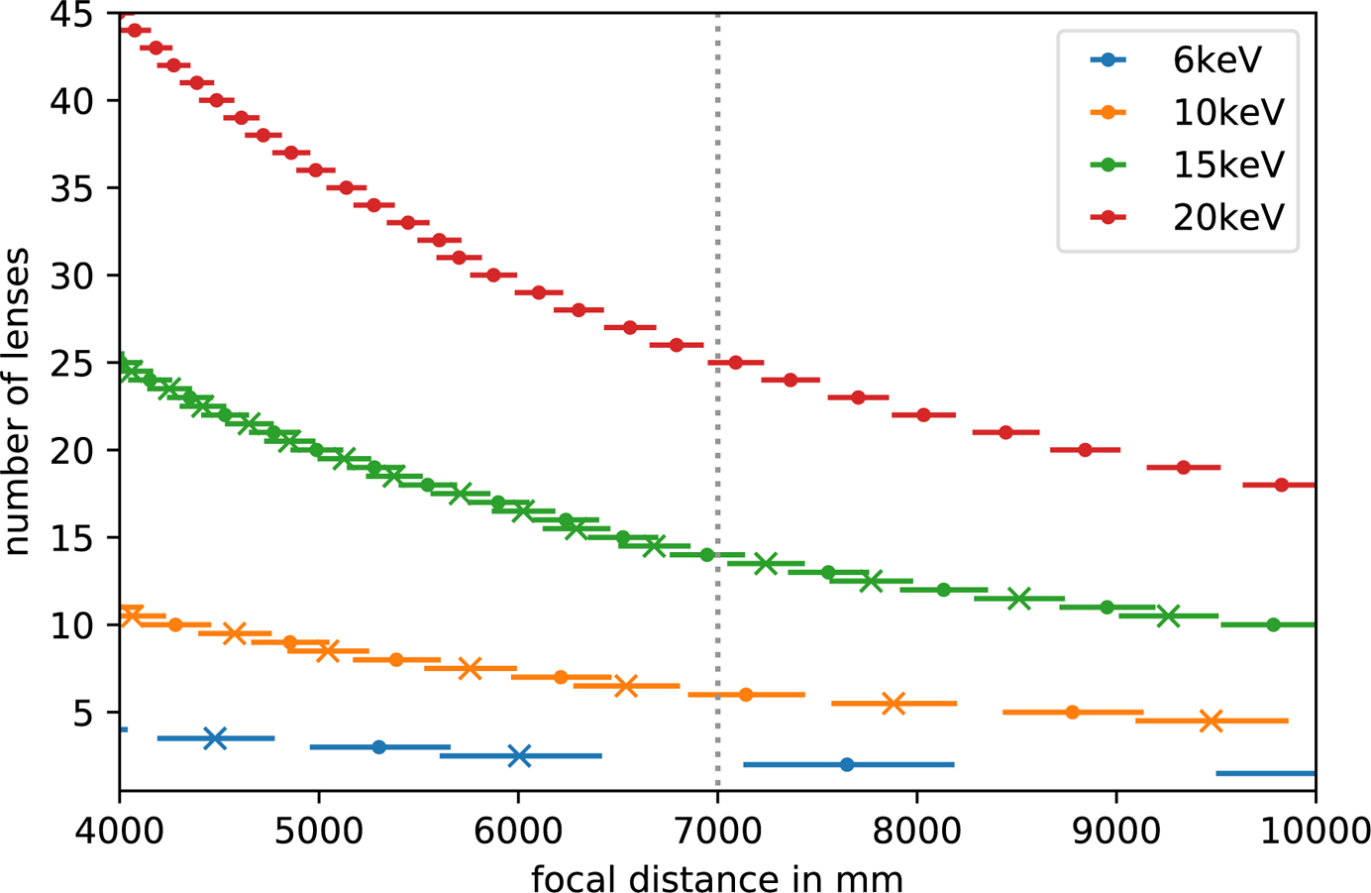
Plot of the number of lenses required for a desired focal distance across the working energy range of the SPB/SFX instrument calculated using ray tracing assuming a parallel beam via the use of additional (pre-focusing) CRLs in the photon delivery tunnels. The dotted vertical line indicates the standard focal distance for the IRD SFX setup. This plot provides a guide for the design of custom (user-designed) experimental setups in the downstream region of the SPB/SFX instrument.

**Figure 4:**
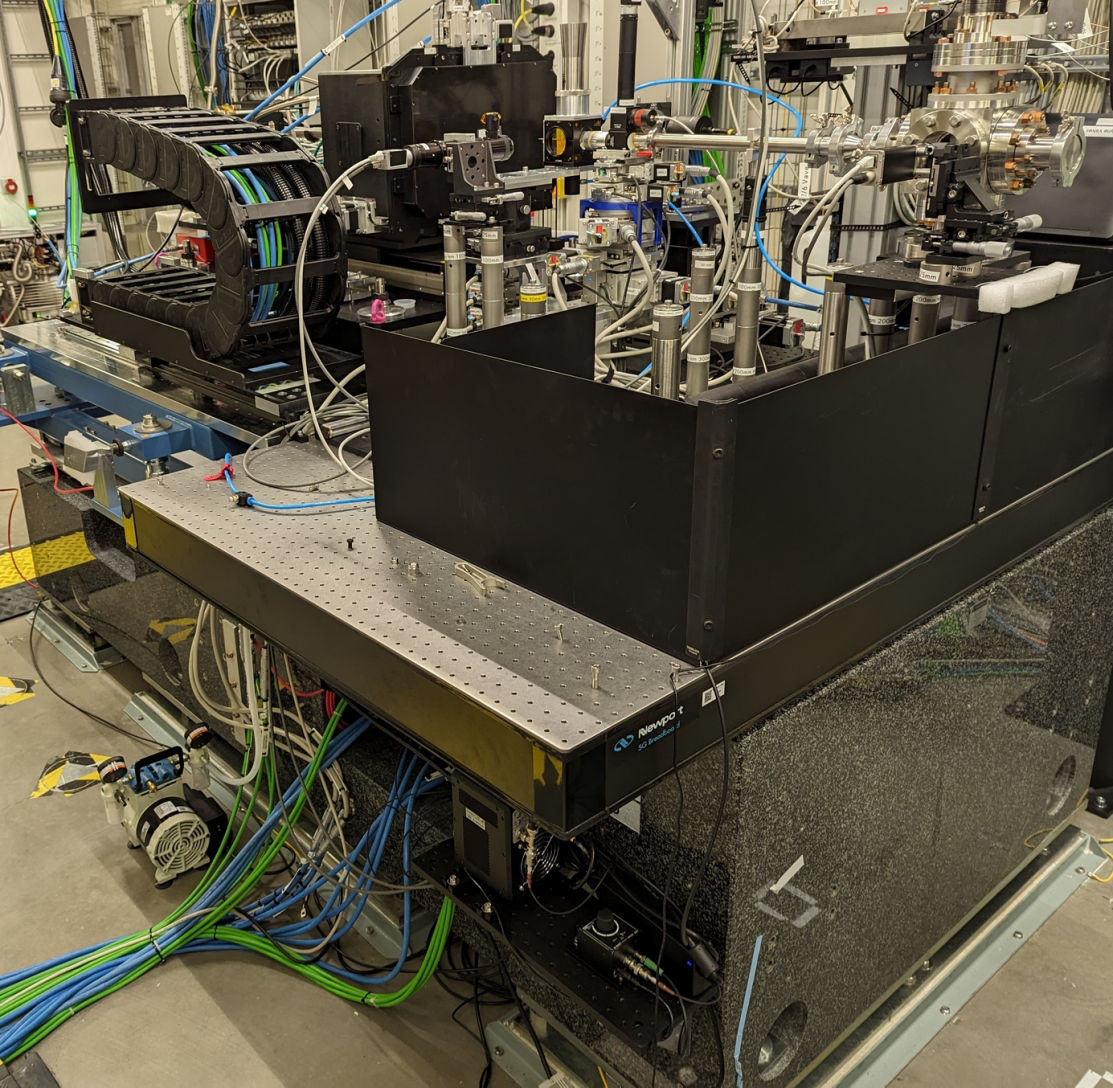
Photograph of the IRD standard SFX setup with the JUNGFRAU 4M detector and Roadrunner sample environment installed on a dedicated granite support for stability.

The CRLs can be used to adjust the beam size from a few millimetres down to the focus size of ∼2.5 µm anywhere between 4 and 10 m from the CRL transfocator across the operational energy range of the SPB/SFX instrument (Fig. 3 ▸). This enables custom (user-designed) experimental devices to be installed for use either upstream or downstream of the dedicated IRD SFX setup (shown in Fig. 4 ▸).

### 2.2. Vacuum to atmospheric pressure interface

The beryllium-based refocusing optics of the SPB/SFX instrument require vacuum (in the 10^−8^ mbar range or lower) for their operation. In order to investigate samples at atmospheric pressure, a safe method of transitioning the beam between ultra-high vacuum and atmospheric pressure is required. The risk of failure of any window cannot be neglected, as catastrophic failure would result in propagation of a shock wave which could damage the fragile and potentially hazardous beryllium CRLs.

To address this risk of failure, a sonic or acoustic delay line (ADL) (Sato *et al.*, 1985 ▸) has been integrated (labelled E in Fig. 1 ▸) as a key element in the protection concept of the vacuum interlock system. Its purpose, in the case of catastrophic failure of the window, is to decelerate the shock wave as it propagates upstream. The overall effect is the introduction of a sufficient buffer of time that allows the detection of the pressure increase in the vicinity of the diamond window and triggers the closure of an upstream shutter before the shock wave can reach the CRL vacuum chamber.

The ADL, developed in close collaboration with the EuXFEL vacuum group (Villanueva, 2017 ▸), consists of a 20 cm diameter vacuum chamber with an overall length of 1 m. It is internally divided into seven separate volumes, each of them connected by means of a central orifice with a diameter of 10 mm. Similar designs were successfully reported in the past (Wolgast & Davis, 1969 ▸). The fast-closing valve has a closing time of 10 ms; the design goal was to generate a delay of at least 30 ms, giving a safety factor of 3, intended to account for uncertainties in the experimental setup.

The device performance was thoroughly tested and the results were successfully validated (Villanueva, 2026 ▸). In order to protect the diamond window against thermally enhanced oxidation (and to minimize background scattering and transmission losses in air), a helium flight path or rough vacuum tube is installed between the diamond separation window and the sample environment interface using an additional Kapton foil.

### 2.3. The JUNGFRAU integrating pixel detector

Eight front-end modules with 320 µm thick silicon sensors have been combined into the four megapixels detector used as standard for SFX in the IRD for the SPB/SFX instrument (Sikorski *et al.*, 2023 ▸). The JUNGFRAU detector’s 16 memory cells can enable an average data collection rate of 160 Hz when 16 pulses are delivered per train for sample delivery methods compatible with replenishment between pulses. This repetition rate provides data collection faster than many other FEL facilities worldwide. However, it is not compatible with efficient simultaneous operation with the IRU as usage at this rate would not provide significant data gains when weighed against the additional expense of complexity and extra risk of sample delivery interruptions.

**Figure 5:**
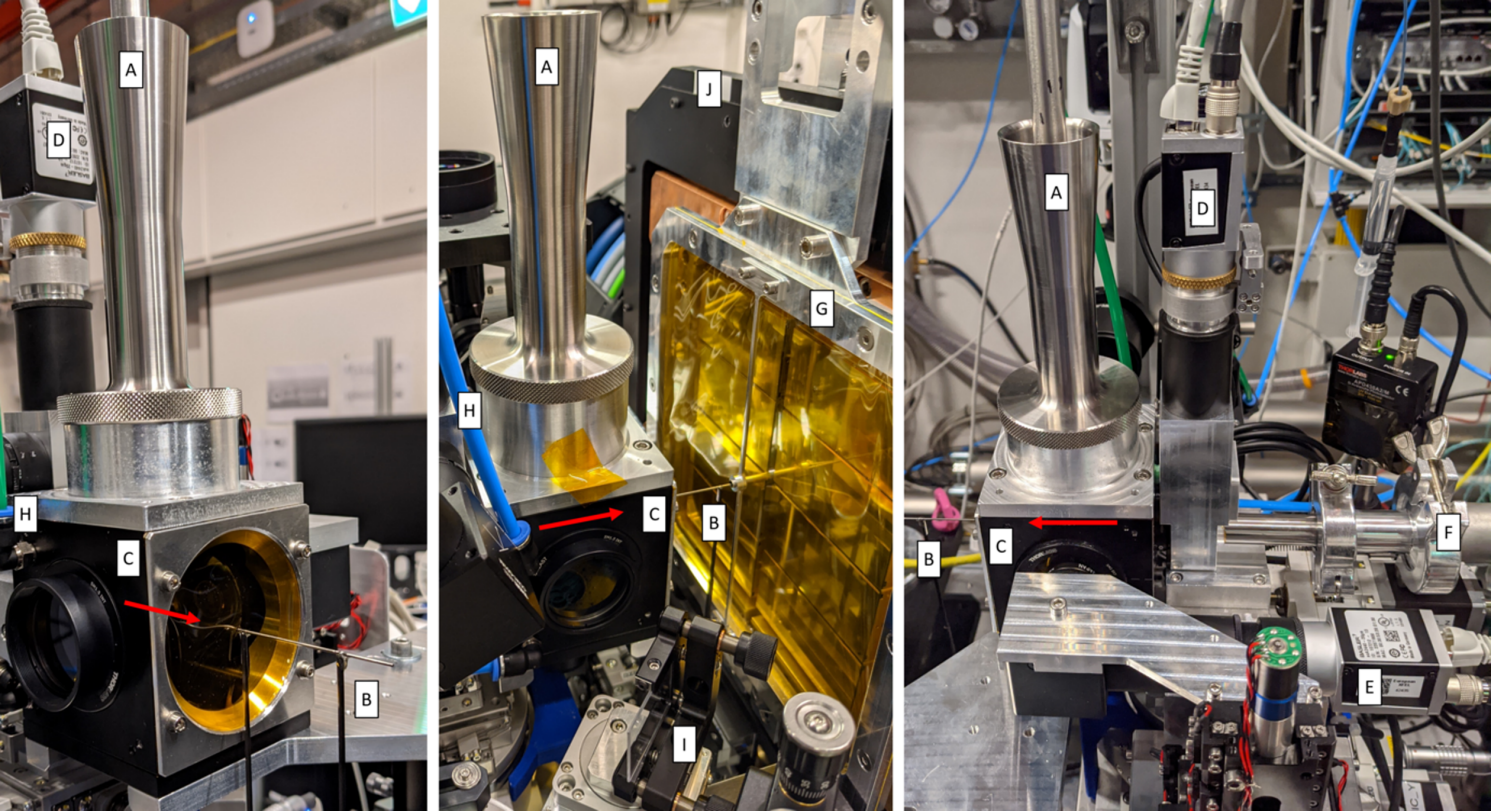
IRD sample environment. (Left) The atmospheric pressure (helium) injection setup mounted on the Roadrunner sample environment, which sits directly in front of (middle) the JUNGFRAU 4M detector, is equipped with (right) inline and 90° microscopes for sample visualization and alignment. Components are labelled as follows: A the injection rod funnel receptor, B the beam guard collimation straw, C the helium atmosphere cube, D the inline microscope camera, E the 90° (side) microscope camera, F the vacuum (or helium) flight tube, G the detector splatter guard with integrated direct beam guard, H the helium input line, I the laser and illumination in-coupling and J the JUNGFRAU 4M detector. Red arrows indicate the beam direction.

The minimum sample to detector distance is 130 mm [with the Roadrunner goniometer (Section 2.5.1)], but a shorter distance of ∼115 mm can be achieved with the small injection helium cube (Fig. 5 ▸). With this configuration, 1.6 Å resolution at the edges of the detector is possible with 12.4 keV X-ray photons, and better than 1.2 Å at 15 keV with the current injector shroud. Plans to enable data collection beyond 0.9 Å (achievable with a larger exit window) are onging as part of an upgraded sample injection environment. Unit cells up to 500 Å are acceptable for data collection at 12 keV with the detector positioned to provide 3 Å resolution at the edge, assuming a minimum spacing of 5 pixels between Bragg peaks. This will also hold for the larger AGIPD-4M detector which would be placed further away to achieve the same resolution.

The JUNGFRAU 4M assembly’s longitudinal motorization allows the sample to detector distance to be adjusted by up to 400 mm, which provides quick access to the interaction point as well as the detector. During a typical experiment, the front-end modules are protected from contamination by sample debris with a Kapton film shield. Several versions of the shield were developed to fit the needs of specific types of measurements ranging from an Al-coated shield, blocking the visible light, to one equipped with a metallic (direct beam scatter) guard straw (labelled B in Fig. 5 ▸) to minimize the low-angle background.

### 2.4. Optical laser systems

A number of optical laser systems are available on the SPB/SFX instrument, delivering pulses ranging from femtosecond to nanosecond duration (Mancuso *et al.*, 2019 ▸; Koliyadu *et al.*, 2022 ▸). The SASE-1 pump–probe laser system provides pulses of duration down to 15 fs centred at 800 nm and sub-pico­second pulses at 1030 nm with a pattern matching that of the FEL, and the typical optical laser beam size at the interaction point is 30–50 µm (Palmer *et al.*, 2019 ▸). In addition, several commercial fixed-wavelength and tuneable nanosecond laser systems operating at lower repetition rates (≤20 Hz) are available. These can be delivered to the IRD either via fibre in-coupling or via free space, depending on the chosen sample delivery method and experiment geometry.

### 2.5. Sample environment

The IRD atmospheric pressure sample environment is designed to be open, accessible and flexible in order to accommodate a wide range of components. Within the 2.5 m between the vacuum out-coupling ADL and detector (bridged by a helium-filled flight path), the main features to accommodate are the interface to the CSS (optical table with 25 mm × 25 mm spaced M6 threaded holes) and the height of the beam (540 mm) from the support. Potentially, any components can be installed with respect to these parameters in order to deliver samples and thereby exploit the X-ray beam.

#### 2.5.1. Sample environments for SFX

As reliable and reproducible data collection is a prerequisite, standardization of the sample environments was a priority. The currently commissioned standard for SFX-type measurements in the IRD is the Roadrunner III sample environment (Roedig *et al.*, 2017 ▸; Tolstikova *et al.*, 2019 ▸) developed at DESY/CFEL. This system incorporates the necessary sample positioning and visualization (inline and 90° microscopes), with the option for through-the-lens laser excitation of the sample (labelled D and E, respectively, in the right-hand image of Fig. 5 ▸), as well as precision alignment for the direct beam scatter guard straw (labelled B in Fig. 5 ▸) to minimize background. Communication between the EuXFEL control system *Karabo* (Hauf *et al.*, 2019 ▸) and the Roadrunner *tango* server allows control from both the *Karabo* and Roadrunner GUIs and logging of motor positions throughout the experiment. This versatile setup is mounted on a large granite block for stability, and it can be optimized for a range of sample delivery options:

(i) Fast-scanning fixed-target goniometer with integrated temperature/humidity-controlled helium chamber.

(ii) Gas-controlled jet-based injection (Fig. 5 ▸).

(iii) High-viscosity injection [*e.g.* lipid cubic phase (LCP)-based media].

(iv) Low-viscosity liquid injection.

**Figure 6:**
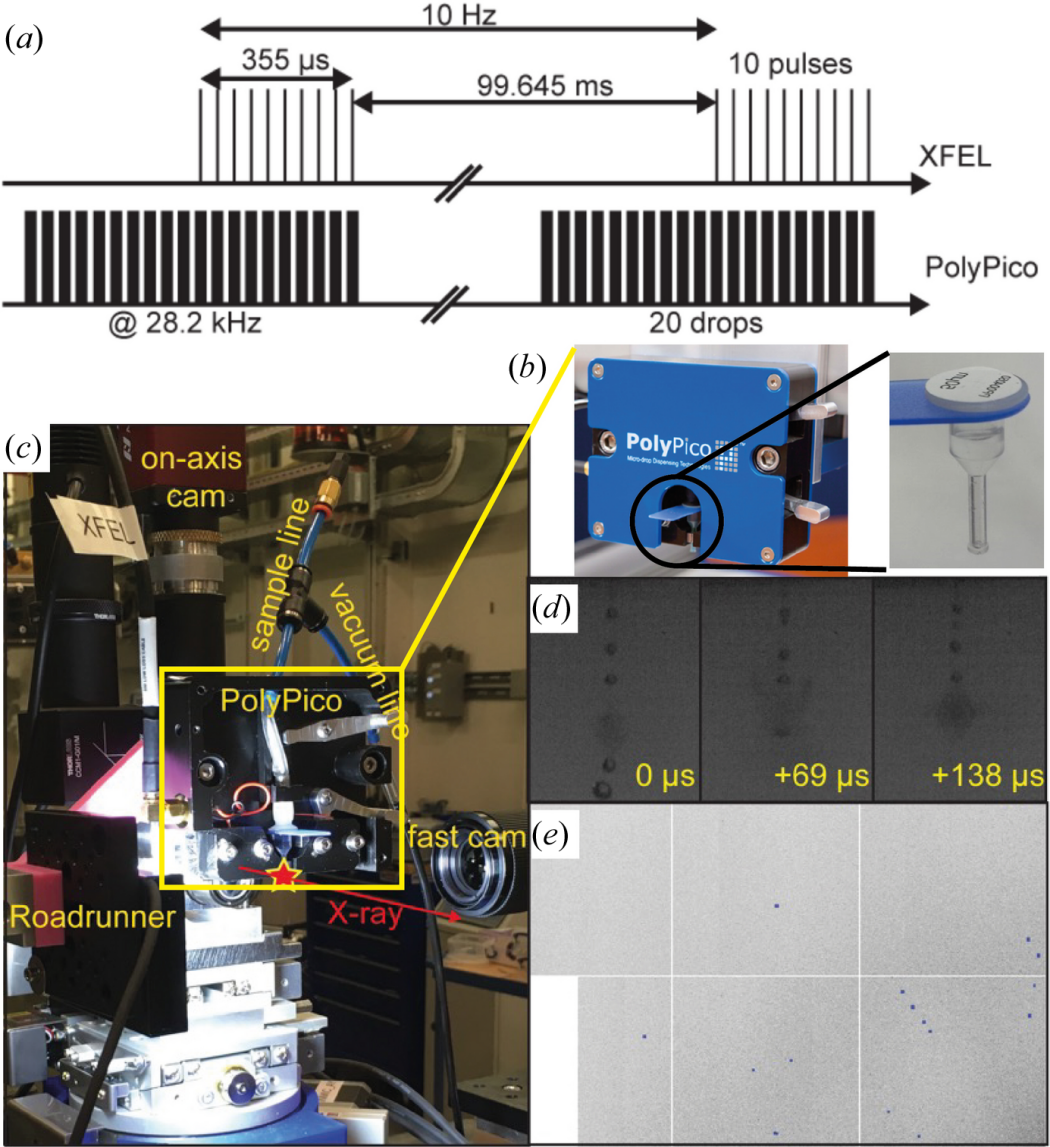
Overview and strategy for on-demand droplet delivery on the EuXFEL SPB/SFX instrument. (*a*) Example of the timing required to execute a 28.2 kHz intra-train injection strategy to achieve 100 Hz overall data collection rate. We dispensed 20 crystal-containing droplets in bursts that started before the arrival of each X-ray pulse train. This stabilized the burst such that the last ten droplets arrived synchronized with the ten X-ray pulses within each 355 µs pulse train. (*b*, *c*) The PolyPico micro-dispensing head and cartridge mounted in the IRD. (*d*) A high-speed video camera observes droplets in the interaction region. This example shows three sequential droplet explosions within the same pulse train. (*e*) Example JUNGFRAU images clearly show diffraction patterns that are consistent with lysozyme microcrystals.

(v) Drop-on-demand acoustic droplet injection (Fig. 6 ▸).

#### 2.5.2. High-speed Roadrunner goniometer for fixed-target experiments

For scanning of fixed-target sample holders, a Roadrunner goniometer (Roedig *et al.*, 2017 ▸) is available for use in the IRD of the SPB/SFX instrument. In consists of a rotation axis aligned in the horizontal direction, which is equipped with a centring stage. On top of the centring stage, a fast scanning stage operated with a linear motor is mounted. This allows high-speed scanning of the fixed-target sample holders in the horizontal direction with scanning speeds of up to 100 mm s^−1^, compatible with kilohertz data collection (Tolstikova *et al.*, 2019 ▸). This is not amenable to scanning within the intra-train pulse structure of the European XFEL and is therefore implemented for data collection at 10 Hz. The goniometer is capable of scanning sample holders with a size of up to 12 mm × 40 mm. The scanning axis is equipped with a magnetic mount enabling quick changeover of a wide variety of solid mounts (chips) for investigation of crystalline samples. This efficient (minimal wastage) sample delivery can achieve very high (>90%) hit rates using pores on the support to position crystals matched with the scanning steps. The goniometer interface makes a seal with the Roadrunner measurement chamber to preserve the helium environment and allow easy access for chip exchange.

Due to the time for measurement and the desire to mount crystals with minimal liquid for optimum data quality, the use of dry helium (required for low scattering background) would result in detrimental dehydration of the sample. The Roadrunner measurement chamber can be continuously flushed with a humid helium stream with a user-defined relative humidity ranging from 70% to 100%. Additionally, the measurement temperature inside the chamber can be freely selected between 273 and 333 K. For permanent monitoring of temperature and relative humidity, the Roadrunner chamber is equipped with four built-in sensors in order to avoid drying of crystals and build up of water caused by condensation in the chamber.

#### 2.5.3. Liquid jet sample delivery

Liquid jets are an important sample delivery method for SFX experiments, especially at megahertz rates. The liquid jet systems at the SPB/SFX instrument are designed to maintain compatibility with established liquid sample delivery systems developed for experiments at FEL facilities while allowing flexibility in nozzle design. More details can be found in the paper by Vakili *et al.* (2022 ▸).

Sample delivery nozzles can be mounted on the goniometer to enable accurate and reproducible alignment, or on a short nozzle rod controlled by the goniometer mounting stages, inserted into the helium environment through a funnel-shaped nozzle rod receptor (labelled A in Fig. 5 ▸) (Schulz *et al.*, 2019 ▸).

#### 2.5.4. Viscous jet sample delivery

Many membrane proteins are crystallized in lipid cubic phase (LCP) and therefore are naturally in a state of high viscosity. Crystals from soluble proteins can also be mixed into viscous carrier media and injected with a high-viscosity extrusion (HVE) jet to improve sample efficiency. For serial crystallography of these kinds of samples, an HVE jet (Weierstall *et al.*, 2014 ▸) can also be installed onto the Roadrunner goniometer. However, in practice the weight of these injector types can easily exceed the limits of the fast scanning stage. To overcome this limitation the nozzle can be mounted on a frame enabling positioning using the *XYZ* motors of the goniometer support tower. A range of injectors and nozzle designs can be used with this standard setup including viscous mixing nozzles (Vakili *et al.*, 2023 ▸).

HVE injection based on hydraulic pressure amplification enables SFX on native membrane proteins (Feld & Frank, 2014 ▸; Neutze *et al.*, 2015 ▸) and has been successfully used in a number of experiments (Sugahara *et al.*, 2015 ▸; Martin-Garcia *et al.*, 2017 ▸; Botha *et al.*, 2015 ▸; Hadian-Jazi *et al.*, 2021 ▸). The flow speed of HVE is not sufficient to provide new sample within the intra-train pulse separation and is thus limited to a single pulse per train. The current achievable repetition rate for single-exposure data collection with LCP injection is therefore 10 Hz at EuXFEL.

#### 2.5.5. Drop-on-demand sample delivery

For biology applications, such as SFX experiments with protein crystals, high sample consumption is a bottleneck even for a high repetition rate XFEL. Ideal sample delivery for SFX experiments at XFELs should be robust and efficient with respect to:

(i) providing a high hit rate,

(ii) consuming minimal sample,

(iii) allowing rapid acquisition of full data sets,

(iv) efficient use of the available X-ray pulses,

(v) being compatible with a variety of different sample types and

(vi) minimizing the time needed to change from one sample to another.

With the aim of addressing all these issues, we have integrated and tested an on-demand piezoelectric droplet ejection strategy (Fig. 6 ▸), in collaboration with the XFEL Hub at Diamond Light Source and a commercial developer of drop-on-demand devices (PolyPico Technologies Ltd, Cork, Ireland). Ongoing development efforts and results using PolyPico injectors at Diamond Light Source, LCLS, SACLA and PAL-XFEL have already been published (Butryn *et al.*, 2021 ▸; Davy *et al.*, 2019 ▸). This sample delivery strategy delivers discrete droplets of solution or microcrystal slurry into the X-ray pulses, synchronized with their arrival and paused during the dark time, thereby conserving sample. The sample volume required for a complete data set (assuming 50 pl droplets, 20% hit ratio) requires only a few microlitres to yield the requisite minimum of several thousands hits. Different-sized droplets (50–150 µm diameter) can be ejected using different inner diameter cartridges [Fig. 6 ▸(*b*)] which are rapidly exchangeable with no cross contamination.

Confirmation of synchronization was obtained using a side-mounted camera with stroboscopic illumination to visualize droplet explosions of sequential drops within the burst [Fig. 6 ▸(*d*)]. The JUNGFRAU detector images clearly show diffraction patterns that are consistent with lysozyme microcrystals [Fig. 6 ▸(*e*)]. Each droplet travels at ∼1.5 m s^−1^ and the explosions did not show any significant effects on subsequent droplets at 28.2 kHz (frequency of the PolyPico delivery). These results demonstrate that the on-demand method using a PolyPico-based delivery strategy can enable sample-efficient data collection to yield SFX structures from only microlitres of crystal slurry.

### 2.6. Data analysis

#### 2.6.1. Online

Online data processing, analysis and visualization are used for monitoring experimental data during acquisition. The EuXFEL control system *Karabo* (Hauf *et al.*, 2019 ▸) includes the visualization of detector images using the online calibration/correction pipeline. Further online analysis with custom tools is available accessing the data via the *Karabo* bridge. Online tools to facilitate standard experiments, such as *OnDA* for SFX, are provided (Mariani *et al.*, 2016 ▸).

#### 2.6.2. Offline processing

The most frequently used offline software for SFX on the SPB IRD is *CrystFEL* (White *et al.*, 2016 ▸). The EuXFEL data analysis group provides optimized processing using the *CrystFEL* suite via the *EXtra-Xwiz* framework (Turkot *et al.*, 2023 ▸). The *EXtra-Xwiz* tool enables automatic data preparation and management of distributed computing on the HPC cluster. Initially developed for AGIPD-1M data, *EXtra-Xwiz* has been extended to support JUNGFRAU 4M data as well.

### 2.7. First results

Measured background scattering (from air) is minimal (on average <2 photons per pixel per frame) at full transmission with helium flow and beam guard (collimation) straws. For comparison, in the absence of helium flow, air scatter on the detector in the range of >20 photons per pixel at low angles with <1% transmission is observed. The optimized designs of the local helium environments (labelled C in Fig. 5 ▸) are essential for successful operation at atmospheric pressure and ensure low background scattering for all standard sample delivery options.

Five sample delivery methods have been tested and are available in the IRD (Section 2.5.1), demonstrating not only feasibility but also the desired flexibility of the downstream interaction region. Strong diffraction could be seen from crystals using each of the presented injection methods. The structures solved from the data and the statistical information obtained during the commissioning of the IRD (see supporting information) gives confidence (to us and to the users) for the continued success of IRD experiments as part of the regular calls for proposals for experiments on the SPB/SFX instrument of the European XFEL.

## 3. Conclusions

The infrastructure for the operation and use of the downstream interaction region on the SPB/SFX instrument has been described, including its X-ray refocusing geometry, sample delivery systems, detection systems and first diffraction data (at atmospheric pressure). Importantly, this demonstrates successful serial crystallography with the CRL-focused beam in the IRD, increasing flexibility and the future availability of experimental time, access, data collection and scientific results obtained on the SPB/SFX instrument at the European XFEL. This allows not only increased efficiency of the SPB/SFX instrument but also the efficient use of precious samples with fixed-target, HVE or drop-on-demand sample delivery.

## 4. Related literature

The following references, not cited in the main body of the paper, have been cited in the supporting information: Dickerson *et al.* (2020 ▸); Garman & Weik (2023 ▸); Liebschner *et al.* (2019 ▸); Perrett *et al.* (2024 ▸); wwPDB Consortium (2019 ▸); Zeldin *et al.* (2013 ▸).

## Supplementary Material

Additional data on lysozyme. DOI: 10.1107/S1600577525008999/yi5181sup1.pdf

## Figures and Tables

**Table 1:** Summary of basic parameters of the downstream region of the SPB/SFX instrument

Parameter	IRD	IRU
Photon energy	6–20 keV max	3–20 keV
	8–16 keV recommended	
Pulse energy (max)	*ca* 1–5 mJ	*ca* 1–5 mJ
Photons per pulse (at instrument)	*ca* 1–5 × 10^12^ photons	*ca* 1–8 ×10^12^ photons
Focal spot size	∼2.5 µm	∼0.2–2.5 µm
Pulse duration	25–100 fs	25–100 fs
Detectors	JUNGFRAU 4M (current)	AGIPD 1M
	AGIPD 4M (planned)	
Detector pixel size	JUNGFRAU 75 µm × 75 µm	AGIPD 200 µm × 200 µm
Single photon sensitivity	Yes	Yes
Detector dynamic range (12 keV)	> 10^4^ photons	> 10^4^ photons
Detector edge resolution (injection setup)	∼1.6 Å (12 keV)	∼1.6 Å (12 keV)
	∼1.2 Å (15 keV)	∼1.2 Å (15 keV)
Detector frame rate:	JUNGFRAU	AGIPD
Single pulse per train operation	10 s^−1^	10 s^−1^
Up to burst mode	10 × 16 (160) s^−1^	10 × 352 (3520) s^−1^
Sample-to-detector distance	0.105 to ∼0.5 m	0.9–5.5 m
Sample delivery options: liquid jet	Up to 1.1 MHz	Up to 1.1 MHz
High viscosity	10 Hz	10 Hz
Fixed targets	10 Hz	10 Hz
Drop-on-demand	10–160 Hz	Not applicable in vacuum
