# Supplementary material for: The Single-Particle, Clusters and Biomolecules and Serial Femtosecond Crystallography instrument of the European X-ray Free-Electron Laser: Interaction Region Downstream at atmospheric pressure (IRD)
Source: J Synchrotron Radiat. 2026 Jan 1;33(Pt 1):198–206. doi: 10.1107/S1600577525008999 (PMC12809452; doi:10.1107/S1600577525008999)
Supplement: Supplementary file 1 [file s-33-00198-sup1.pdf]

JOURNAL OF SYNCHROTRON RADIATION

Volume 33 (2026)

Supporting information for article:

The single-particle, clusters and biomolecules, and serial femtosecond crystallography instrument of the European X-ray Free-Electron Laser: interaction region downstream at atmospheric pressure (IRD)

Adam Round, Piere Aller, Richard J. Bean, Johan Bielecki, Agata Butryn, Nicholas Devenish, Raphael de Wijn, Thomas Dietze, Katerina Doerner, Fabio Dall'Antonia, Pontus Fischer, Gabriele Giovanetti, Sebastian Guenther, Huijong Han, Vincent Hennicke, Chan Kim, Yoonhee Kim, Marco Kloos, Jayanath C P Koliyadu, Gabriel Leen, Romain Letrun, Luis Lopez Morillo, Allen M. Orville, Tim Pakendorf, Marco Ramilli, Nadja Reimers, Patrick Reinke, Juan Sanchez-Weatherby, Tokushi Sato, Robin Schubert, Joachim Schulz, Cedric Signe Takem, Marcin Sikorski, Prasad Thute, Fabian Trost, Oleksii Turkot, Patrik Vagovič, Mohammad Vakili, Raul Villanueva Guerrero, Henry N. Chapman, Alke Meents, Serguei Molodtsov, Sakura Pascarelli, Thomas Tschentscher and Adrian P. Mancuso

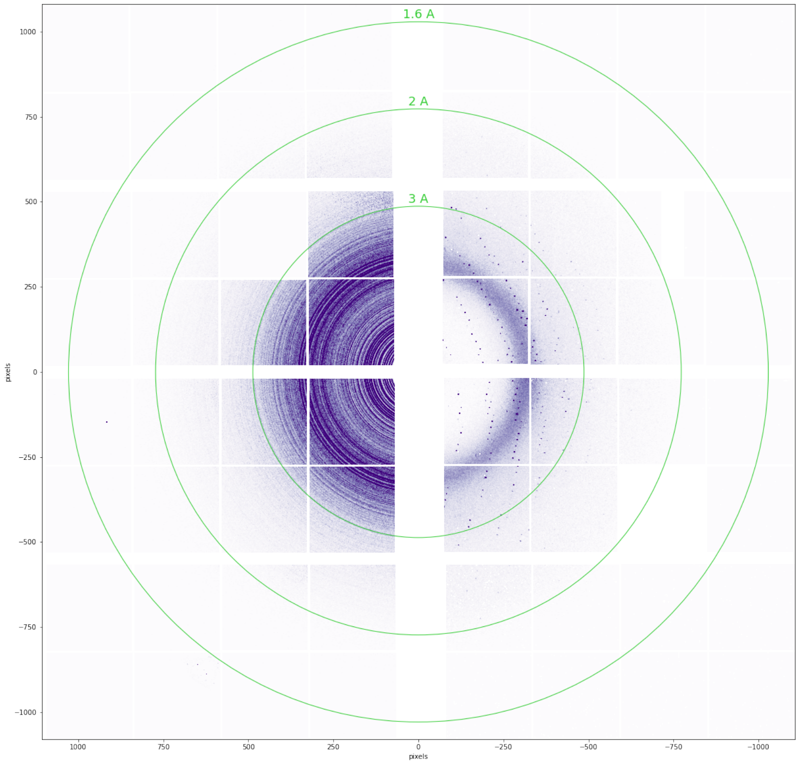

Fig. S1. Diffraction pattern (dataset 1) from Lysozyme crystals collected with the JUNGFRAU 4M detector using LCP injection. Left side, sum of the indexed peaks from dataset 1. Right side, an example of an individual frame. Calculated resolution at edge of Jungfrau 4M detector at 12.4keV is 1.6 Å extending beyond 1.4 Å in the corners. The prominent (diffuse) ring scattering at around 4.5 Å resolution arises from monoolein.

S1.**Supplementary data**

5.0.1. Lysozyme datasets Two lysozyme datasets were collected at 12.25 and 9.61 keV using LCP injection and the Jungfrau 4M detector. A diffraction pattern from dataset 1 is presented in Fig. S1; data collection details and statistics are detailed in Table S1. Raw data were calibrated using the calibration pipeline from the EuXFEL

and processed with the Xwiz-workflow ((Turkot *et al.*, 2023)), Crystfel v0.10.1 (White *et al.*, 2016) and Phenix v1.21 ((Liebschner *et al.*, 2019)). Raw and processed data are available upon request to open.data@xfel.eu under the doi 10.22003/XFEL.EU-DATA-002697-00 (runs 4 to 8, 34 to 45 and 51 for dataset 1) and 10.22003/XFEL.EU-DATA-900201-00 (runs 128 to 181 for dataset 2). The refined structures are deposited in the Protein Data Bank (PDB) ((wwPDB consortium, 2019)) under the numbers 9RRL and 9RRK respectively.

The overall X-ray doses were calculated using RADDOSE-3D ((Zeldin *et al.*, 2013)) version 5.0 and the extension RADDOSE-XFEL ((Dickerson *et al.*, 2020)). Average calculated doses for both datasets are below the dose limit determined for room-temperature data collected at synchrotrons ((Garman & Weik, 2023)).

Table S1. *Data quality statistics and figures of merit for two Lysozyme datasets collected at the IRD*

**Data Collection**	**Dataset 1 (PDB 9RRL)**	**Dataset 2 (PDB 9RRK)**
Injection method	LCP	LCP
Data collection rate	10 Hz	10 Hz
X-ray energy (keV)	12.25	9.61
Frames count	199446	257657
Hit rate (%)	24.75	31.23
Indexing rate (%)	182.05	143.06
Overall indexing rate (%)	45.05	44.68
Nr. of indexed crystals	89859	115110
Space group	P 43 21 2	P 43 21 2
Cell dimensions (Å)	79.30, 79.30, 38.60	79.30, 79.30, 38.60
Cell dimensions (°)	90, 90, 90	90, 90, 90
Resolution	18.25 - 1.70 (1.76 - 1.70)	21.98 - 1.85 (1.92 - 1.85)
Rsplit	7.89 (119.00)	11.20 (15.46)
CC1/2 (%)	99.36 (30.32)	97.59 (95.66)
CC* (%)	99.84 (68.21)	99.39 (98.88)
SNR	9.08 (0.75)	11.69 (7.20)
Completeness	100.00 (100.00)	100.00 (100.00)
Multiplicity	1024.7 (739.6)	1000.7 (567.2)
**Refinement**		
No. reflections	26 534 022	20 130 406
Rwork/Rfree	18.67/23.93	16.63/20.50
Bond lengths (Å)	0.010	0.009
Bond angles (°)	1.046	0.922
RADDOSE-XFEL average dose exposed region (kGy)	23.7 ± 0.6	57.3 ± 1.5

5.0.2. Lysozyme preparation Lysozyme crystals with sizes of 5–6 µm were prepared in aqueous solution as previously described ((Perrett *et al.*, 2024)). Prior to embed-ding the lysozyme crystals in the lipidic cubic phase (LCP), the LCP containing the lysozyme crystal storage buffer was prepared as follows: First, the lysozyme crystal storage solution was diluted with water to 42% of its original concentration. The diluted solution was then transferred into a gas-tight glass syringe. A second syringe was filled with melted monoolein. The volume ratio of the two solutions was fixed at 2:5 for the diluted lysozyme crystal storage solution and monoolein, respectively. The two syringes were connected using a syringe coupler, and the solutions were mixed by moving the plungers back and forth. Once the mixture became transparent, the entire sample was transferred into one syringe, and the other syringe was removed. The filled syringe was then connected to a third syringe containing lysozyme crystal pellet which contained 10% of the volume relative to the prepared LCP in the other syringe. The crystals were mixed into the prepared LCP by moving the plunger until the pellet was evenly distributed throughout the syringe.

5.0.3. Lysozyme preparation and delivery The LCP containing the lysozyme crystals was delivered as described here ((Weierstall *et al.*, 2014)). The injector system was driven by a high-performance liquid chromatography (HPLC) pump (Shimadzu LC-20AD) with an average delivery of 0.5 uL/min and a Helium gas stream with an average of 10 mg/min was used to stabilize the delivery.
